# Can uric acid blood levels in renal transplant recipients predict allograft outcome?

**DOI:** 10.1080/0886022X.2021.1969246

**Published:** 2021-08-25

**Authors:** Ofer Isakov, Bhanu K. Patibandla, Doron Shwartz, Eytan Mor, Kenneth B. Christopher, Tammy Hod

**Affiliations:** a Department of Internal Medicine “T”, Tel Aviv Souraski Medical Center, Tel Aviv University, Tel Aviv, Israel; b Division of Pulmonary and Critical Care Medicine, Oregon Health and Science University, Portland, OR, USA; c Department of Nephrology, Souraski Medical Center, Tel Aviv University, Tel Aviv, Israel; d Renal Transplant Center, Sheba Medical Center, Tel Aviv University, Tel Aviv, Israel; e Renal Division, Brigham and Women’s Hospital and Harvard Medical School, Boston, MA, USA; f Department of Nephrology, Sheba Medical Center, Tel Aviv University, Tel Aviv, Israel

**Keywords:** Hyperuricemia, renal allograft function, renal allograft survival, mortality

## Abstract

**Background:**

Hyperuricemia is common after renal transplantation, especially in those receiving calcineurin inhibitors. Little, however, is known about the relationship between uric acid (UA) levels and allograft outcome.

**Methods:**

We conducted a retrospective single-center analysis (*N* = 368) in order to assess UA blood levels post-transplant association with allograft outcome. For this study, a median serum UA level of all measured UA levels from 1 month to 1 year post renal transplantation was calculated.

**Results:**

Patients were divided into 2 groups based on the median UA level measured between 1 and 12 months post-transplant. Those with median UA level ≥ 7 and ≥ 6 mg/dL (*N* = 164) versus median UA level < 7 and < 6 mg/dL for men and women respectively (*N* = 204) had lower GFR values at 1, 3 and 5 years posttransplant (mean GFR ± SD of 43.4 ± 20.6 and 58 ± 19.9 at 3 years post-transplant, *p* < 0.001). In multivariate models, UA levels were no longer significantly associated with renal allograft function. In a multivariate cox proportional hazard model, UA level was found to be independently associated with increased risk for death-censored graft loss (HR of 1.3, 95% CI 1.0–1.7, *p* < 0.05 for every increase of 1 mg/dL in UA level).

**Conclusion:**

Hyperuricemia was found to be associated with increased death- censored graft loss but not with allograft function. Increased UA levels were not found to be an independent predictor of long-term allograft function despite the known association of hyperuricemia with the progression of cardiovascular and renal disease.

## Introduction

Post-renal-transplant hyperuricemia is common with a prevalence of 15.5% to 84% [1]. Numerous risk factors have been reported including reduced allograft function, use of calcineurin inhibitors (CNI) primarily cyclosporine, diuretic use, obesity and advanced age [1–3]. Experimental and epidemiological evidence suggests that uric acid (UA) and hyperuricemia play a role in cardiovascular and renal disease [4–8]. Several studies have linked hyperuricemia with an increased risk of hypertension (HTN) and chronic kidney disease (CKD) progression by inducing renal inflammation [9,10], oxidative stress [11,12], endothelial dysfunction [13], decreased nitric oxide production and activation of the renin-angiotensin-aldosterone system (RAAS) [14–17]. Serum UA has also been associated with coronary artery calcification and carotid intimal thickening [18,19]. Induced hyperuricemia in rats leads to the development of HTN and renal interstitial fibrosis. Normalization of serum UA with Allopurinol decreased the extent of fibrosis [16]. In another study, renal pathology in rats who had oxonic acid added to their diet to induce hyperuricemia showed afferent arteriole thickening with renal cortical vasoconstriction and glomerular HTN leading to interstitial inflammation and fibrosis. These pathologic changes were not seen in rats maintained on normal serum UA levels [17]. The adverse effects of UA may be further potentiated in kidney transplant recipients (KTR) on CNI as hyperuricemia appears to exacerbate interstitial fibrosis in rats treated with Cyclosporine [18].

The introduction of Cyclosporine in the 1980s led to dramatic reductions in the rate of early acute rejection and greatly improved 1-year graft survival [20,21]. Despite that, there has not been significant improvement in long-term patient and graft survival [22]. In spite of the markedly reduced mortality from cardiovascular disease (CVD) among transplant recipients compared to end-stage kidney disease (ESKD) patients on dialysis, heart disease is still the major cause of death in KTR. In fact, progressive allograft fibrosis and death with a functioning graft account for more than 80% of graft loss after the first-year post-transplant, with 38.2% of the deaths with graft function due to a known CVD [23,24]. Therefore, effort should be made to identify novel risk factors for the poor patient and graft outcomes. There has been a limited number of studies looking at the association of hyperuricemia with long-term patient and allograft outcomes with conflicting results such that the role of UA in the pathogenesis of renal transplant dysfunction and graft failure remains unclear. The need for the treatment of hyperuricemia in KTR is also controversial. A key problem is a relation between UA and graft function. Hyperuricemia may be a consequence of reduced GFR in KTR or may contribute to reduced allograft function. Given the possible link between hyperuricemia, HTN, CVD and progression of CKD as well as renal allograft dysfunction we sought to unravel the association of serum UA level post-renal transplantation to patient and allograft outcome and specifically determine whether hyperuricemia is directly harmful to the renal allograft or a marker of other causal risk factors.

## Methods

### Study design

This is a retrospective analysis of data gathered from the Research Patient Data Registry (RPDR) system at Brigham and Women’s Hospital (BWH), in addition to data we collected from the clinical records. Biochemical data were retrieved in an automated fashion from the laboratory database and clinical data from electronic medical records. This study was approved by the local ethics committee.

### Patients

All patients, who underwent kidney transplantation at BWH between January 2000 and September 2013 were evaluated for inclusion in the study and constituted a population of 856 transplants. Exclusion criteria were: patients without serum UA levels from 1 month–1 year post-transplant (*n* = 449) including those whose graft failed in the first year post-transplant (*n* = 39).

### Immunosuppression

Induction therapy consisted of either basiliximab (Simulect Novartis Pharma, Basel, Switzerland), or Thymoglobulin (Genzyme, Boston, MA). All patients received corticosteroids, beginning with a single preoperative bolus of 500 mg methylprednisolone intravenously, with gradual tapering to 20 mg oral prednisone at postoperative day 5. If the patient was a candidate for early steroid withdrawal (ESW), the prednisone was continued at 20 mg PO daily until tacrolimus levels have reached the therapeutic goal (>8 ng/ml) and then stopped. If the patient did not achieve therapeutic tacrolimus trough concentrations by postoperative day (POD) 12 the prednisone dose was tapered down below 20 mg/day. In patients who were not candidates for ESW, prednisone was tapered down to 5 mg/day within 2 months of transplant.

All patients were started orally on 1 gm mycophenolate mofetil (Cellcept, Roche Pharma, Basel, Switzerland) or 720 mg of mycophenolate sodium (Myfortic, Novartis Pharma) preoperatively; followed by 2 × 1 gm/day or 2 × 720 mg/day, respectively, with tapering of the dosage depending on individual needs and/or side effects.

CNI therapy was started post-transplantation, using mostly tacrolimus, aiming for trough levels of 8–10 ng/mL in the first 3 months, and 5–8 ng/mL thereafter with reduction of trough goals if patients had side effects.

Biopsy proven acute rejection (BPAR) was treated with three doses of intravenous methylprednisolone (500 mg daily) on consecutive days. All patients received pneumocystis jiroveci pneumonia prophylaxis (sulfamethoprimcotrimoxazole or atovoquone). Prophylaxis for cytomegalovirus (CMV) disease using gancyclovir, (Cytovene Roche Pharma) up to 2003 and then valganciclovir (Valcyte, Roche Pharma) was started in all patients.

### Laboratory data

The following biochemical post-transplantation variables were retrieved from the RPDR system: serum creatinine, mean serum LDL and HDL from 1 month to 1 year post-transplant and Tacrolimus 12 h trough blood levels. Median serum UA values from 1 month to 1 year post-transplant were calculated using all samples available from this period. High median serum UA level was defined in the present study as a median serum UA level ≥7 mg/dL in men and ≥6 mg/dL in women, consistent with previous epidemiologic studies [25–27]. We chose to look at serum UA levels not earlier than 1 month post-transplant in order to prevent the confounding effect of delayed or slow graft function (SGF) and the early use of diuretics on UA blood levels. Apart from the median UA, the mean and the lowest registered values over this period were also recorded. As the mean values yielded similar results to median UA only the results based on the median will be reported here.

The presence or absence of SGF was determined based on whether serum creatinine on postoperative day 5 was above 3 mg/dL (presence of SGF) or ≤3 (absence of SGF).

Median tacrolimus 12 h trough blood levels were calculated based on all trough levels available from 1 month to 1-year post-transplant in order to associate tacrolimus trough with serum UA within the same period. Both the median and mean trough levels of tacrolimus from 1 month to year post-transplant were recorded. As those gave similar results, only the results based on the median are reported here.

Serum creatinine levels were retrieved at different time points: 3 months post-transplant (3 months ± 2 weeks post-transplant), 1 year post-transplant (1 year ± 3 months post-transplant), 3 years post-transplant (3 years ±3 months post-transplant) and 5 years post-transplant (± 6 months). Glomerular filtration rate was calculated according to the following CKD Epi formula: GFR= 141* min (Scr/k, 1)*^α^* * max (Scr/k, 1)^−1.209^ * 0.993^Age^ * 1.018 [if female] * 1.159 [if black]. [*K* = 0.7 if female, 0.9 if male; *α*= −0.329 if female, −0.411 if male; min = the minimum of Scr/k of 1; max = the maximum of Scr/k or 1].

### Study assessments

Based on the electronic patient medical records, details on the following relevant items were collected: age, gender, race, transplant type, type of induction treatment, medical history {specifically the history of hypertension (HTN), heart failure (HF), cardiovascular disease (CVD) or pretransplant diabetes}, new-onset diabetes after transplant (NODAT), rejection episodes from 1 month to 1-year post-transplant, use of any loop diuretics, thiazides, Allopurinol, prednisone, angiotensin-converting enzyme inhibitors (Ace inh.), angiotensin receptor blockers (ARB’s), beta-blockers (BB’s) and statins from 1 month to 1-year post-transplant. The nature of the original kidney disease was noted as either ‘diabetic nephropathy’, or ‘other’.

### Statistical analysis

Descriptive statistics were expressed as percentages for categorical data or mean (±SD) for continuous variables. Differences in baseline characteristics between the groups were tested using Chi-square for the categorical variables or t-test for the continuous variables.

We analyzed subgroups based on the availability of serum creatinine at 1, 3 and 5 years post-transplant.

A multivariable linear regression analysis was constructed to analyze the association between median UA levels and allograft outcome while adjusting for covariates and potential confounders. The variables used in the multivariate analysis were chosen based on their clinical relevance, i.e., those with possible interaction with UA blood levels and/or influence on allograft function.

In order to account for patient-level variability, a repeated measures analysis was performed using a linear mixed-effects model with a random intercept calculated for each patient ID number. Median UA level, years post-transplant, and the confounders were used as the fixed effects. *p*-values are provided *via* Satterthwaite’s degrees of freedom method. Mixed-effects model analysis was performed using the R packages *lme4* and *lmerTest*.

We assessed the risk of graft loss and patient’s death based on UA level using Kaplan-Meier curves and the log-rank test, followed by multivariable-adjusted Cox proportional hazards models adjusted for covariates and potential confounders.

A p-value of less than 0.05 was considered statistically significant. Data were analyzed using R version 3.5.2. (http://www.r-project.org).

## Results

Three hundred sixty-eight transplants (208 men and 160 women) of the original population of 856 transplants fulfilled the inclusion criteria. Patients were divided into 2 groups based on the median UA level measured between 1 and 12 months post-transplant. 164 (45%) had median UA level from 1 month to 1-year post-transplant ≥7 and ≥6 (high UA group) and 204 (55%) had median UA level <7 and <6 in men and women respectively (low UA group).

### Univariate comparison high UA versus low UA groups

Pre and post-transplantation clinical and biochemical parameters comparing the high and low UA groups are presented in Table 1. The mean age was 52.2 ± 13.8 in the high UA group and 51.4 ± 13.9 in the low UA group. 12.8% of patients in the high and 10.3% in the low UA group had ESKD secondary to diabetes.

**Table 1.** Patient characteristics.

	High UA (*N* = 164)	Low UA (*N* = 204)	*p-*value
UA *N*	1.60 (1.96)	1.44 (1.37)	0.354
Age (years)	52.2 (13.8)	51.4 (13.9)	0.553
Gender			
Males	84 (51.2)	124 (60.8)	0.083
Females	80 (48.8)	80 (39.2)	
Race/ethnicity			
White	99 (60.4)	134 (65.7)	0.398
Black	40 (24.4)	38 (18.6)	
Other	25 (15.2)	32 (15.7)	
ESRD d/t diabetes	21 (12.8)	21 (10.3)	0.557
Transplant type			
LRD/LURD	70 (42.7)	138 (67.6)	<0.001
SCD	37 (22.6)	31 (15.2)	
DCD/ECD	57 (34.8)	35 (17.2)	
Type of induction			
Thymoglobulin	111 (67.7)	135 (66.2)	0.394
Simulect	51 (31.1)	62 (30.4)	
None	2 (1.2)	7 (3.4)	
TACRO_median (ng/mL)	7.69 (1.72)	7.92 (1.68)	0.208
Rejection episodes	0.03 (0.17)	0.01 (0.1)	0.15
Medical history			
HTN	156 (95.1)	175 (85.8)	0.005
CVD	107 (65.5)	79 (38.4)	<0.001
HF	66 (40.0)	71 (34.8)	0.335
Pre transplant DM	55 (33.3)	66 (32.5)	0.898
NODAT	16 (9.7)	8 (3.9)	0.041
SGF	86 (52.4)	61 (29.9)	<0.001
Use of diuretics			
PO loop diuretics	49 (29.7)	29 (14.3)	0.001
IV loop diuretics	31 (18.8)	15 (7.4)	0.002
PO thiazides	18 (11)	8 (3.9)	0.016
Use of other meds			
Allopurinol	6 (3.6)	17 (8.4)	0.104
Prednisone	77 (47.0)	79 (38.7)	0.139
Ace inh/ARBs	17 (10.4)	7 (3.4)	0.014
BBs	78 (47.6)	71 (34.8)	0.018
Statins	54 (32.9)	50 (24.5)	0.096
Lab tests			
LDL mean mg/dL	87.8 (30.8)	89.1 (29.4)	0.689
HDL mean mg/dL	46.9 (14.2)	52.0 (16.7)	0.003
GFR_3M (ml/min)	43.3 (15.1)	58.4 (17.8)	<0.001

138 patients (67.6%) in the low UA group had a living transplant compared to only 70 KTR (42.7%) in the high UA group (*p* < 0.001). Medical history of HTN and CVD was more prevalent in the high compared to the low UA group (*p* = 0.005 and *p* < 0.001 respectively). 9.7% of KTR in the high compared to 3.9% of patients in the low UA group had NODAT (*p* = 0.044). Due to the association of diuretics with hyperuricemia, we looked at the use of loop diuretics as well as disothiazides from 1 month to 1-year post-transplant. The use of loop diuretics was higher in the high compared to the low UA group (*p* < 0.001 for PO loop diuretics and *p* = 0.002 for the use of intravenous loop diuretics). More KTR used disothiazides in the high compared to the low UA group (*p* = 0.016) as well as other medications including prednisone for maintenance immunosuppression, Ace inh/ARB’s and BB’s. Dyslipidemia with low mean HDL cholesterol levels was more prevalent in the high compared to the low UA group (mean HDL of 46.9 ± 14.2 mg/dL versus 52.0 ± 16.7 mg/dL in the high and low UA respectively, *p* = 0.003). No statistically significant differences were observed for the mean LDL cholesterol level between the groups. 52.5% of the patients in the high UA group had SGF compared to 29.9% in the low UA group (*p* < 0.001). Baseline allograft function derived from GFR at 3 months post-transplant was lower in the high compared to the low UA group (43.3 ± 15.1 versus 58.4 ± 17.8; *p* < 0.001). For all other non-significant differences between variables in the two groups see Table 1.

### Univariate outcome comparison high UA versus low UA

GFR was found to be significantly lower in the high compared to the low UA group at 1 year (45.9 ± 17.30 versus 60.8 ± 19.7, *p* < 0.001), 3 years (43.4 ± 20.6 versus 58.0 ± 19.9, *p* < 0.001) and 5 years post-transplant (41.8 ± 21.4 versus 53.6 ± 23.1, *p* = 0.009) (see Table 2).

**Table 2.** Graft function in the high and low UA groups.

	High UA	*N*	Low UA	*N*	*p-*value
GFR 1 year (ml/min)	45.9 (17.3)	148	60.8 (19.7)	172	<0.001
GFR 3 years (ml/min)	43.4 (20.6)	95	58.0 (19.9)	94	<0.001
GFR 5 years (ml/min)	41.8 (21.4)	50	53.6 (23.1)	51	0.009

### Linear regression analysis with UA and GFR as continuous variables

We performed a multivariable linear regression analysis to establish the change in GFR in relation to UA blood level. UA level was not found to be an independent predictor of GFR at 1, 3 and 5 years post-transplant. For every increase in baseline allograft function of 1 mL/min GFR increased by 0.8 [0.7–0.9], 0.54 [0.37–0.71] and 0.7 [0.4–1.00] ml/min at 1, 3 and 5 years post-transplant respectively (*p* < 0.01).

Use of loop diuretics from 1 month to 1-year post-transplant was found to be associated with reduced GFR at 1-year post-transplant but had no significant association to allograft outcome at 3 and 5 years post-transplant. The presence of CVD was associated with a worse graft function at 3 years post-transplant as GFR decreased by 6.7 [12.5–1.00] ml/min (*p* < 0.05) but not at 1 or 5 years post-transplant. An increase in median CNI trough level was associated with an increased GFR at 1-year post-transplant but not at 3 or 5 years post-transplant. All other variables including age, gender, race, transplant type, induction therapy, medical history of HTN or heart failure (HF), tacrolimus coefficient of variation, pre-transplant diabetes and the presence of SGF were not significantly associated with differences in GFR at 1, 3 and 5 years post-transplant (Table 3).

**Table 3.** Change in GFR in relation to UA blood level using multivariate linear regression model.

	GFR 1 year post Tx	GFR 3 years post Tx	GFR 5 years post Tx
	Study population (*n* = 320)	Study population (*n* = 189)	Study population (*n* = 101)
	Mean (SD)	Mean (SD)	Mean (SD)
Increase in UA of 1 mg/dL	–0.56 (–1.49, 0.36)	–1.48 (–3.2, 0.26)	0.43 (–2.5, 3.4)
Age (increase in 1 year)	–0.03 (–0.15, 0.08)	–0.15 (–0.36, 0.06)	0.04 (–0.36, 0.43)
Female (vs. male)	1.14 (–1.76, 4.04)	0.75 (–4.7, 6.23)	2.2 (–6.4, 10.8)
Race			
White	Reference	Reference	Reference
Black	1.00 (–2.8, 4.79)	–3.05 (–10.0, 3.9)	–6.29 (–18.1, 5.5)
Other	–0.86 (–4.6, 2.9)	–3.6 (–10.1, 2.87)	–4.98 (–16.9, 7.0)
Transplant type			
DCD/ECD vs. LRD/LURD	1.03 (–3.3, 5.3)	5.6 (–2.3, 13.5)	–1.16 (–14.4, 12.1)
SCD vs. LRD/LURD	–0.2 (–4.41, 4.0)	–0.38 (–7.36, 6.6)	4.37 (–6.2, 14.9)
Induction			
Thymoglobulin	Reference	Reference	Reference
Simulect	1.28 (–1.7, 4.2)	–0.73 (–6.1, 4.6)	–4.8 (–15.6, 6.0)
No induction	–2.0 (–15.5, 11.5)	–3.7 (–22.8, 15.3)	13.7 (–14.7, 42.0)
Medical history			
Hypertension	0.5 (–4.6, 5.6)	7.7 (–2.3, 17.8)	4.8 (–9.9, 19.6)
Heart failure	–1.1 (–4.3, 2.1)	–3.1 (–9.2, 3.1)	–6.8 (–17.0, 3.3)
Cardiovascular Disease	–1.6 (–4.6, 1.4)	–6.7 (–12.5, −1.0)*	–8.1 (–17.3, 1.0)
Pre Transplant Diabetes	–1.2 (–4.3, 1.9)	–4.5 (–9.9, 0.98)	–6.8 (–15.4, 1.8)
Median CNI trough level	0.99 (0.12, 1.8)*	1.4 (–0.04, 2.9)	0.8 (–1.47, 3.1)
Tacro coefficient of variation	–0.01 (–0.1, 0.08)	0.03 (–0.13, 0.19)	0.13 (–0.17, 0.43)
Use of loop diuretics	–6.5 (–10.1, −2.9)**	–2.2 (–8.9, 4.5)	0.8 (–10.3, 11.9)
SGF vs. no SGF	0.3 (–3.4, 4.0)	–2.32 (–9.0, 4.36)	3.9 (–8.0, 15.7)
GFR_3M increase in 1 ml/min	0.8 (0.7, 0.9)**	0.54 (0.37, 0.71)**	0.7 (0.4, 1.0)**

Note: The results shown in the table were derived from 4 separate linear regression models; each of them adjusted for the following covariates: age, race, gender, transplant type, induction therapy, medical history, median and coefficient of variation CNI trough level, use of loop diuretics, pre-transplant diabetes, presence of SGF and baseline GFR. *p < 0.05; **p < 0.01.

### Mixed effects model

In order to analyze all the collected measures together while accounting for patient-level measurement variability and dependence, a repeated measures analysis was performed using a linear mixed-effects model. In this analysis, GFR was found to be associated with time post-transplant (Every 1-year post-transplant GFR decreased by 1.8 mL/min; CI 2.0–1.6; *p* < 0.01), medical history of CVD (GFR decreased by 6.3 mL/min in renal transplant recipients with CVD compared to those without it; CI 10.2–2.4; *p* < 0.01), black race (GFR decreased by 5.5 mL/min; CI 10.4–0.7; *p* < 0.05 in blacks compared to white) and with baseline allograft function (GFR increased by 0.6 mL/min for every increase in baseline GFR of 1 mL/min; CI 0.5–0.72; *p* < 0.01). All other variables including UA blood level, age, gender, transplant type, induction therapy, medical history of HTN, HF and pre-transplant diabetes, median and coefficient of variance for tacrolimus 12 h trough levels, use of loop diuretics and the presence of SGF were not significantly associated with GFR over time (Table 4). The variation in GFR between patients was estimated to be 13.83 mL/min.

**Table 4.** Mixed effects model.

	GFR
	Mean (SD)
Years post transplant	–1.8 (–2.0, −1.6)**
Increase in UA of 1 mg/dL	–0.73 (–1.9, 0.4)
Age (increase in 1 year)	0.02 (–0.13, 0.17)
Female (vs. male)	–0.64 (–4.4, 3.1)
Race	
White	Reference
Black	–5.5 (–10.4, −0.7)*
Other	–3.5 (–8.5, 1.4)
Transplant type	
DCD/ECD vs. LRD/LURD	2.2 (–3.27, 7.7)
SCD vs. LRD/LURD	0.9 (–4.4, 6.2)
Induction	
Thymoglobulin	Reference
Simulect	–1.37 (–5.2, 2.4)
No induction	3.6 (–12.9, 20.1)
Medical history	
Hypertension	–2.2 (–9.0, 4.7)
Heart Failure	–2.6 (–6.7, 1.6)
Cardiovascular disease	–6.3 (–10.2, −2.4)**
Pre-transplant diabetes	–2.6 (–6.5, 1.4)
Median CNI trough level	0.87 (–0.2, 1.97)
CNI coefficient of variation	–0.09 (–0.2, 0.02)
Use of loop diuretics	–3.1 (–7.6, 1.5)
SGF vs. no SGF	0.5 (–4.2, 5.2)
GFR_3M increase in 1 ml/min	0.6 (0.5, 0.72)**

Note: The results shown in the table were derived from 4 separate linear regression models; each of them adjusted for the following covariates: age, race, gender, transplant type, induction therapy, medical history, median and coefficient of variance CNI trough level, use of loope diuretics, pre-transplant diabetes, presence of SGF and baseline GFR. *p < 0.05; **p < 0.01.

### Graft and patient survival analyses

Patients were followed up for up to 120 months post-transplant with a mean follow-up time of 50.1 months. During this period, 21 cases in the high UA group (14.5%) and 8 in the low UA group (4.5%) lost their grafts (*p* = 0.004). 14 (9.7%) and 13 (7.3%) patients died in the high and low UA groups respectively (*p* = 0.59).

**Figure 1.**
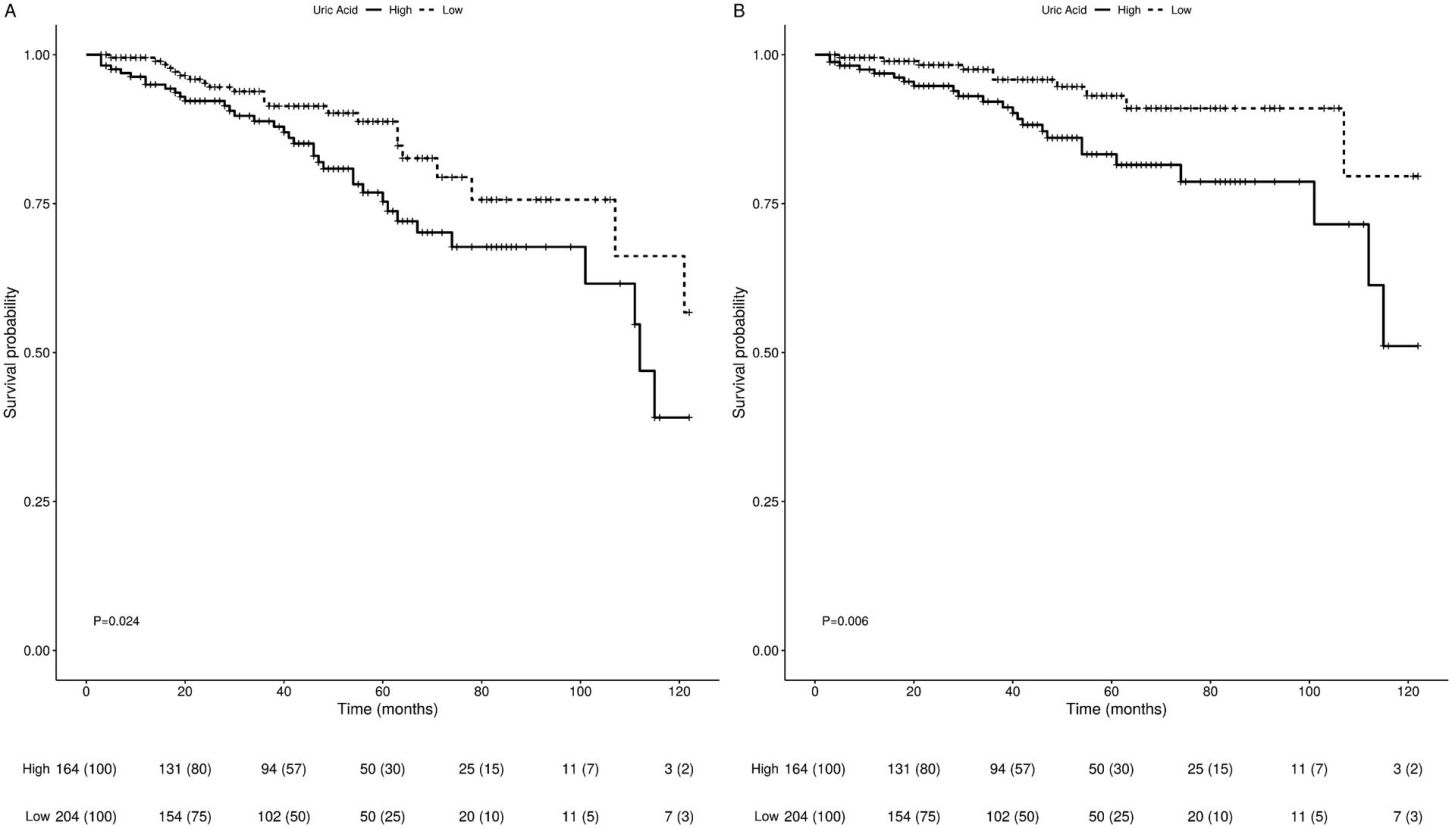
Univariate Kaplan–Meier curves for overall graft survival (A), death-censored graft survival (B). (A), (B) show comparisons of survival between the high and low UA groups with log-rank *p*-values of 0.024 and 0.006 respectively.

**Figure 2.**
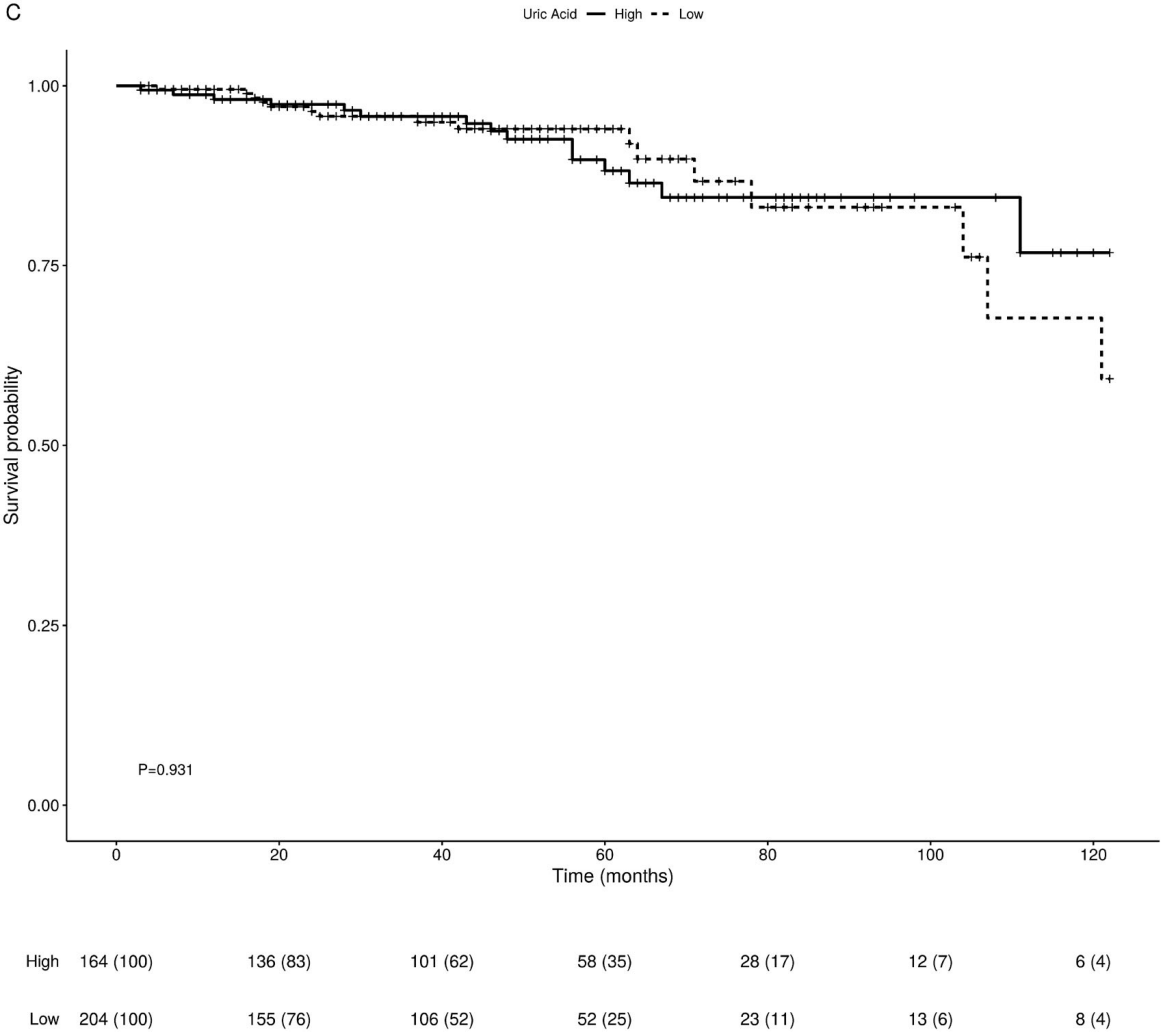
Univariate Kaplan–Meier curve for patient survival showing comparisons of survival between the high and low UA groups with log-rank *p*-values of 0.931.

In a univariate Kaplan–Meier survival analysis rate of graft failure including death with a functioning graft over time was significantly higher in the high compared to the low UA groups (*p* = 0.024). When death with a functioning graft was excluded from the analysis, we observed a greater variance between the two groups (*p* = 0.006) (see Figure 1). Patient survival was not found to be statistically different between the high and low UA groups (*p* = 0.931) (see Figure 2).

In a multivariate Cox regression hazard model adjusted for age, gender, race, transplant type, induction therapy, medical history, median and coefficient of variance CNI trough level, use of loop diuretics, pre-transplant diabetes, presence of SGF and baseline allograft function increased UA level post-transplant was found to be a significant independent predictor for only death-censored graft loss during the study period (hazard ratio [28] of 1.3, 95% CI 1.0–1.7, *p* < 0.05 for every increase of 1 mg/dL in UA level). Among the other variables included in the analysis black race, coefficient of variance CNI trough level and baseline graft function were found to be independently associated with death-censored graft loss (see Table 5).

**Table 5.** Multivariate cox regression hazard model for death-censored graft loss.

	HR
	Mean (95% CI)
Years post-transplant	0.99 (0.96, 1.02)
Increase in UA of 1 mg/dL	1.3 (1.0, 1.7)*
Age (increase in 1 year)	0.99 (0.96, 1.02)
Female (vs. male)	0.8 (0.32, 1.9)
Race	
White	Reference
Black	3.28 (1.2, 9.1)*
Other	2.2 (0.7, 7.2)
Transplant type	
DCD/ECD vs. LRD/LURD	0.5 (0.1, 1.7)
SCD vs. LRD/LURD	0.4 (0.1, 1.4)
Induction	
Thymoglobulin	Reference
Simulect	1.8 (0.78, 4.31)
No induction	1.05 (0.07, 14.91)
Medical history	
Hypertension	1.5 (0.17, 13.5)
Heart failure	2.06 (0.8, 5.5)
Cardiovascular disease	1.24 (0.46, 3.34)
Pre-transplant diabetes	0.7 (0.3, 1.75)
Median CNI trough level	1.0 (0.8, 1.3)
CNI coefficient of variation	1.04 (1.02, 1.1)**
Use of loop diuretics	0.94 (0.36, 2.4)
SGF vs. no SGF	0.65 (0.2, 1.95)
GFR_3M increase in 1 ml/min	0.96 (0.93, 1.00)*

Note: The results shown in the table were derived from 4 separate multivariate Cox regression hazard models; each of them adjusted for the following covariates: age, race, gender, transplant type, induction therapy, medical history, median and coefficient of variance CNI trough level, use of loope diuretics, pre-transplant diabetes, presence of SGF and baseline GFR. *p < 0.05; **p < 0.01.

## Discussion

Whether UA has a direct effect on long-term renal allograft function and survival or is just a marker of allograft function is still subject to debate. We sought to shed light on this important topic.

We demonstrated that KTR with increased median UA level from 1 month to 1-year post-transplant has more HTN, CVD, NODAT and dyslipidemia manifested by lower HDL levels. These patients also use more diuretics, antihypertensive medications such as BB’s and RAAS inhibitors and have a higher prevalence of deceased donor renal transplants and DCD/ECD renal transplants compared to KTR with a lower UA level during that period. Despite these findings, UA level was not found to be independently associated with renal allograft function up to 5 years post-transplant in a multivariate and a mixed effect model analyses adjusted for median and coefficient of variance CNI trough levels, use of diuretics, presence of SGF and baseline allograft function. We could not establish an independent association of UA level to allograft function in subgroups of KTR who only had a living-related transplant, in those with severe hyperuricemia (UA≥8 mg/dL) or with good allograft function at 1-year post-transplant (GFR> 60 mL/min). In addition, we could not show an independent association to allograft function when looking at UA level at 3 months post-transplant or median UA level from 1 to 6 months post-transplant (results not shown). Median UA level from 1 month to 1-year post-transplant was found to be independently associated with increased risk for death-censored graft loss over time, despite the absence of association to allograft function.

In CKD, renal excretion of UA is decreased, resulting in hyperuricemia. Interstitial accumulation of sodium urate may induce deterioration of the disease. A summary of results from 24 studies in which most reports identified hyperuricemia as an independent risk factor for CKD progression concluded that decreasing UA levels in hyperuricemic CKD patients may attenuate CKD progression [29]. Moreover, a large retrospective study with 16,186 patients divided into 3 groups (patients receiving no urate-lowering therapy, patients with poor and good adherence to therapy) found that patients who reached target serum UA levels of ≤6 mg/dL achieved a 37% reduction in CKD progression [30]. However, contrary evidence has been suggested and the causal relationship between UA and renal disease has not been established. In the MDRD (Modification of Diet in Renal Disease) Study, a randomized controlled trial was designed to test low versus usual protein intake on CKD progression in participants with eGFR of 13–55 mL/min/1.73m^2^, no association was observed between UA and CKD progression [31].

Studies have raised the hypothesis that elevated UA is associated with progressive renal allograft dysfunction. A correlation of hyperuricemia with the rate of decrease in renal allograft function over time, independent of initial eGFR has been observed. Hyperuricemia was also found predictive of cardiac events in this population [32]. In a retrospective cohort study of 212 LDKT recipients, a significant independent association between mean UA level during the first 6 months post-transplant and long-term graft survival was found. UA level was also found to be independently associated with graft function at 1-year post-transplant [24]. In another retrospective cohort study of 307 patients, an association between hyperuricemia at 6 months post-transplant and the development of new cardiovascular events and chronic allograft nephropathy (CAN) was found, especially in patients with eGFR of less than 50 mL/min, suggesting that hyperuricemia might have an additive effect in the context of decreased allograft function, increasing the risk for the development of CVD and CAN in KTR [27]. New-onset gout was found to be an independent predictor of death and transplant loss [2]. A retrospective cohort study suggested that a low to normal serum UA level within the first year and 1–5 years post-transplant might be an independent factor for better renal allograft outcomes in the long term [33]. Conversely, in a sub-study of the symphony trial with 1645 participants, no significant effect of UA concentrations on renal allograft function was found [34]. UA concentration was not observed as an independent risk factor for renal allograft outcome in models that accounted for graft function as a time-varying confounder, suggesting that UA is not an independent risk factor for graft failure [26]. In a large prospective randomized kidney transplant clinical trial, examining the impact of UA concentrations on outcomes revealed that a high UA concentration among patients following kidney transplantation is not an independent risk factor for cardiovascular outcomes or transplant survival [35]. Based on these conflicting results one cannot distinguish whether hyperuricemia is simply a consequence of reduced allograft function or is causative.

Several mechanisms for the effect of UA on native renal and renal graft outcome have been proposed. The association between hyperuricemia and cardiovascular risk, HTN, microalbuminuria, diabetes mellitus, obesity and CKD has been well known for many years [5]. UA was pointed as one of the mediators of endothelial dysfunction [13,16] and inhibition of xanthine oxidase can ameliorate endothelial dysfunction, potentially improving long-term outcomes [36]. In patients with CKD stage 3, regression of left ventricular hypertrophy has been demonstrated with Allopurinol treatment for 9 months [37]. In addition, UA has been identified to have possible effects on the immune system and proinflammatory pathways. UA may aid in the recognition of apoptotic cells by dendritic cells and the activation of CD8 cells [38]. A high intracellular UA concentration facilitates protein kinase and transcription of proinflammatory cytokines and chemokines and provokes proximal tubular dysfunction with the release of inflammatory chemokines [39].

The role of hyperuricemia in CKD progression as well as in renal allograft function and outcome remains controversial. This is partly related to the difficulty in isolating the relation between UA and GFR, because of the multiplicity of confounding effects of factors, such as a change in GFR over time, use of diuretics, antihypertensive medications and immunosuppressive therapy in the KTR population. The association of hyperuricemia to CVD, HTN and other risk factors make it even more complex as multiple closely related factors which play a role cannot be easily taken into account simply by multivariate analysis. Although many of these associations were detected in our study by univariate analysis, adjustment for covariates rendered some associations nonsignificant. In fact, only the presence of CVD was found to be an independent predictor of long-term renal allograft function both in multivariate analysis and in a mixed effect model. This finding may be due to the awareness of immunosuppressive exposure reduction post-transplantation as well as addressing cardiovascular risk factors in the modern era. Consistent with previous reports, the present study also shows that increased UA level is associated with an increased risk of long-term graft failure [40]. Interestingly, the CNI coefficient of variance was found to be independently associated with death censored graft loss in a multivariate analysis similar to previous publications which showed the association of higher variability of tacrolimus trough level with BK nephropathy, acute rejection [41] and graft survival [42].

Certain limitations should be considered when interpreting the results of this study. The study is an observational study performed retrospectively showing the association (but not the causative relationships) between the primary variables of interest and outcome. Even though we adjusted for numerous variables, residual confounding parameters may still exist. The long follow-up period and standardization of treatment practices limit confounding. This is a retrospective study, which often deliberates a choice of immunosuppressive agents, possibly leading to inclusion bias. It does not however abolish the relationship between UA level to graft function and outcome. We believe that increased UA level exerts its negative effect on renal allograft function *via* its association with cardiovascular risk factors and with CVD and is largely a marker of reduced allograft function. Our observation of increased UA level post-renal transplantation with lower quality kidney donors (deceased donors and DCD/ECD as opposed to living donors) with possibly reduced tubular function and impaired ability to excrete UA supports this hypothesis. However, our finding of an independent association of increased UA to death-censored graft loss may indicate that UA level by itself is a risk factor for adverse allograft outcomes. It is possible we could show UA level association to allograft survival since the follow-up time in survival analysis is longer (up to 10 years post-transplant). Survival analysis takes into consideration overall follow-up time and so patients that died during the follow-up period are included in the survival analysis but are excluded from the multivariate analysis of the years following the death. In addition, in the multivariable analysis, we are comparing mean GFR, and since the number of patients with graft loss is fairly small it is possible that their effect on the overall mean is not significant enough.

Until larger prospective randomized controlled studies that allow the adjustment of confounding variables are accomplished the role of UA in progressive renal disease, as well as CVD in the transplant population, will stay debatable. In the meantime, hyperuricemia remains a marker for progressive renal allograft dysfunction and CVD after renal transplantation. Considering the link between UA level, traditional CVS risk factors and CVD, lowering UA level to minimize these risk factors may be beneficial for improving graft outcomes. Minimizing the use of diuretics and cyclosporine and avoiding purine-rich foods and alcohol may also be effective strategies to decrease the serum UA level in KTR.

## Abbreviations

UA: uric acid
HTN: hypertension
CKD: chronic kidney disease
RAAS: renin angiotensin aldosterone system
KTR: kidney transplant recipients
BPAR: biopsy proven acute rejection
CNI: calcineurin inhibitor
ESW: early steroid withdrawal
NODAT: new onset diabetes after transplantation
POD: post-operative day
RPDR: research patient data registry
SGF: slow graft function
